# Oligomerization of DNA replication regulatory protein RADX is essential to maintain replication fork stability

**DOI:** 10.1016/j.jbc.2022.101672

**Published:** 2022-02-02

**Authors:** Taha Mohamed, Madison B. Adolph, David Cortez

**Affiliations:** Department of Biochemistry, Vanderbilt University School of Medicine, Nashville, Tennessee, USA

**Keywords:** genome stability, DNA repair, dimerization, fork protection, DNA combing, RPA, RADX, cxorf57, RAD51, fork reversal, BSA, bovine serum albumin, EV, empty vector, HU, hydroxyurea, OB, oligosaccharide-binding, RPA, replication protein A

## Abstract

Genome integrity requires complete and accurate DNA replication once per cell division cycle. Replication stress poses obstacles to this process that must be overcome to prevent replication fork collapse. An important regulator of replication fork stability is the RAD51 protein, which promotes replication fork reversal and protects nascent DNA strands from nuclease-mediated degradation. Many regulatory proteins control these RAD51 activities, including RADX, which binds both ssDNA and RAD51 at replication forks to ensure that fork reversal is confined to stalled forks. Many ssDNA-binding proteins function as hetero- or homo-oligomers. In this study, we addressed whether this is also the case for RADX. Using biochemical and genetic approaches, we found that RADX acts as a homo-oligomer to control replication fork stability. RADX oligomerizes using at least two different interaction surfaces, including one mapped to a C-terminal region. We demonstrate that mutations in this region prevent oligomerization and prevent RADX function in cells, and that addition of a heterologous dimerization domain to the oligomerization mutants restored their ability to regulate replication. Taken together, our results demonstrate that like many ssDNA-binding proteins, oligomerization is essential for RADX-mediated regulation of genome stability.

The human genome is replicated with high fidelity once per cell division cycle. A highly regulated ensemble of proteins involved in DNA synthesis, chromatin deposition, DNA repair, and replication stress responses ensure replication fork stability and completion of genome duplication in a timely manner.

Fork reversal is one of the mechanisms of replication stress tolerance that facilitates the repair or bypass of DNA damage and other replication stresses (1, 2). RAD51, the recombinase in homology-directed double-strand break repair, works in cooperation with ATP-dependent motor proteins like SMARCAL1, ZRANB3, HLTF, and FBH1 to catalyze fork reversal (3, 4, 5, 6, 7, 8). While reversal can be a replication stress tolerance mechanism, it also generates a DNA structure that is a substrate for both endo- and exo-nucleases that could yield double-strand breaks or degradation of the nascent strands (9, 10, 11, 12). Many fork protection mechanisms, including BRCA2-stabilized RAD51 nucleofilaments, prevent excessive nuclease activity at reversed forks (11).

Too little or too much fork reversal and RAD51 function can be deleterious to genome stability. Furthermore, there are competing pathways of replication stress tolerance such as translesion bypass synthesis and repriming that may be preferred in some circumstances (1, 13). Therefore, RAD51 and fork reversal are tightly regulated. RADX participates in this regulation to ensure genome stability (14, 15, 16, 17, 18).

RADX is an ssDNA-binding protein with similarity to the large subunit of replication protein A (RPA) (16). RADX regulates RAD51 at ongoing and stalled forks. RADX inactivation in the absence of any added replication stress causes fork slowing and collapse which can be rescued by inactivating RAD51 (16).

RADX competes with RAD51 for ssDNA, directly binds RAD51, stimulates RAD51 ATPase activity, promotes RAD51 nucleofilament disassembly, and inhibits RAD51 recombinase functions (15). Furthermore, single-molecule studies show that RADX condenses ssDNA to prevent RAD51 filament assembly (19). The RADX interaction with RAD51 is essential for RADX cellular functions at replication forks (15). While in unchallenged conditions, RADX inhibits RAD51-mediated fork reversal, it is important to promote fork reversal in cells exposed to high levels of replication stress (17). The exact molecular basis for this switch in activity is unclear but may be traced back to a function of RADX in destabilizing RAD51 nucleofilaments in both unstressed and stressed conditions.

In addition to acting as a regulator of RAD51, the interplay between RADX and RPA on ssDNA is important for replication integrity. Ectopic overexpression of RPA exacerbates phenotypes of RADX inactivation; meanwhile mild RPA depletion rescues fork speed and prevents fork collapse upon RADX loss (18).

RADX is predicted to contain at least three oligonucleotide/oligosaccharide-binding domains (OB-fold) domains, OB-1, OB-2, and OB-3 (16). Unlike RPA, there is no evidence that RADX forms hetero-oligomers with additional subunits. At least one of these OB-fold domains (OB-2) binds ssDNA (16). The RADX OB-3 domain contains a surface required for direct interaction with RAD51 (15). RADX was predicted to contain two structured domains in its C-terminal half, labeled domain 4 and domain 5. However, the functions of these RADX regions have not been investigated.

Here, we show that RADX acts as a homo-oligomer. RADX oligomerizes through more than one interface one of which is in predicted domain 5. Furthermore, we find that RADX oligomerization is essential for its genome maintenance activities during DNA replication.

## Results

### RADX forms homo-oligomers

**Figure 1:**
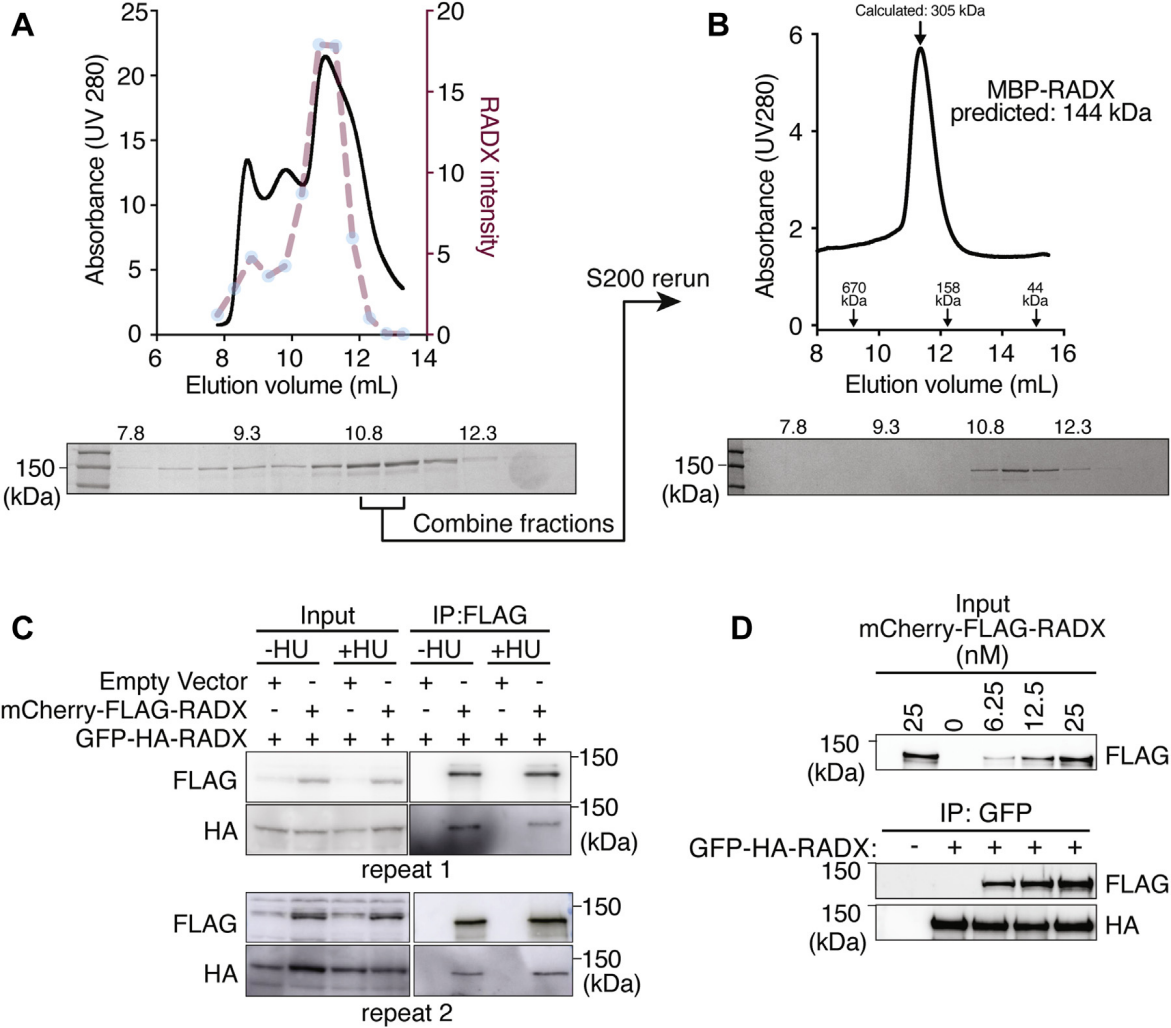
**RADX oligomerizes *in vitro* and in cells.***A*, MBP-RADX was purified and applied to a Superdex 200 Increase 10/300 GL column while measuring UV absorbance at 280 nm. The elution profile is shown as *solid line*. Eluted fractions were also analyzed by SDS-PAGE, and the quantified RADX abundance in those fractions is superimposed as a *dashed-line*. *B*, the fractions containing the highest concentrations of RADX were combined and reapplied to the size-exclusion column. The approximate molecular weight of eluted proteins was determined using the molecular weight standards shown on the *graph*. *C*, either empty vector or RADX (tagged with mCherry-FLAG) expression vector were cotransfected with GFP-HA-RADX into HEK293T cells. FLAG immunoprecipitation from cell lysate was followed by SDS-PAGE and immunoblotting. *D*, purified recombinant mCherry-FLAG RADX was mixed with purified GFP-HA-RADX at the indicated concentrations. GFP-immunoprecipitated proteins were separated by SDS-PAGE and immunoblotted as indicated. HU, hydroxyurea; MBP, maltose-binding protein.

While purifying maltose-binding protein (MBP)-tagged RADX protein expressed in insect cells, we found that concentrated protein elutes from a gel filtration column over a broad range of fractions, with a peak corresponding approximately to the size of a dimer (Fig. 1*A*). Pooling these fractions and reapplying to the column yielded a single peak of RADX elution at an apparent molecular weight corresponding to approximately 305 kDa, which is twice the predicted molecular weight of MBP-RADX (Fig. 1*B*). Thus, we hypothesized that RADX forms dimers or higher order oligomers.

To test this hypothesis, we coexpressed m-Cherry-FLAG-RADX and GFP-HA-RADX in HEK293T cells and found that they are coimmunoprecipitated from cell lysates (Fig. 1*C*). Treating the cells with hydroxyurea (HU) to induce replication stress before lysis did not reproducibly alter the amount of the interaction. This experiment was performed in the presence of DNase to rule out that the interaction is mediated by DNA.

To determine if the interaction is direct, we purified GFP-HA-RADX and mCherry-FLAG-RADX proteins separately and then mixed increasing concentrations of the mCherry-FLAG-RADX with GFP-HA-RADX. Immunoprecipitating GFP-HA-RADX with GFP antibodies also coprecipitated mCherry-FLAG-RADX (Fig. 1*D*), indicating that RADX forms homo-oligomers.

### RADX oligomerization is mediated by more than one interaction interface

**Figure 2:**
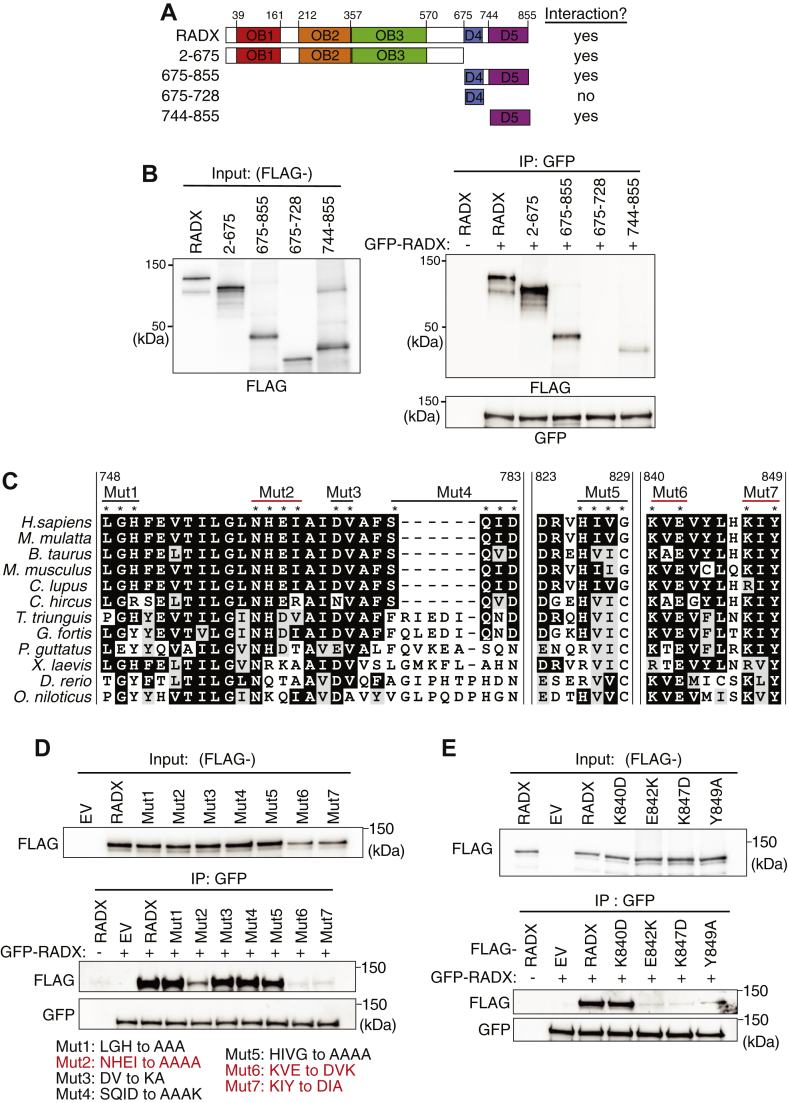
**RADX oligomerizes through two separable motifs.***A*, schematic domain diagram of full-length RADX and RADX fragments. *B*, purified mCherry-FLAG fused to full-length RADX or to RADX fragments were mixed with purified GFP-HA-RADX. GFP-immunoprecipitated proteins were separated by SDS-PAGE and immunoblotted as indicated. *C*, multiple sequence alignment of RADX using Clustal Omega. The depicted regions include human residues 748 to 783, 823 to 829, and 840 to 849. *D*, purified mCherry-FLAG or mCherry-FLAG-RADX or RADX mutants were mixed with purified GFP-HA-RADX. GFP-immunoprecipitated proteins were separated by SDS-PAGE and immunoblotted. *E*, purified mCherry-FLAG alone or fused to WT RADX or to RADX mutants were mixed with purified GFP-HA-RADX. GFP-immunoprecipitated proteins were separated by SDS-PAGE and immunoblotted as indicated. EV, empty vector; OB, oligosaccharide-binding.

To map the region of RADX that mediates oligomerization, we expressed and purified RADX fragments as mCherry-FLAG tagged proteins and assessed whether they can interact with full length RADX tagged with GFP-HA. These RADX fragments were designed based on a previous structure prediction that suggested RADX contains three OB domains and two structured domains termed domain 4 and domain 5 (Fig. 2*A*) (16). We found that RADX may contain more than one oligomerization interface, because nonoverlapping N-terminal (2–675) and C-terminal fragments (675–855) interact with full length RADX (Fig. 2*B*). Domain 5 (744–855) but not domain 4 (675–727) interacts with full length RADX (Fig. 2*B*).

### Mutations that disrupt RADX oligomerization do not impair RAD51 binding or localization to replication forks

To gain more insight into RADX oligomerization, we asked which residues within domain 5 participate in the RADX interaction with itself. Using sequence conservation and predicted secondary structure, we designed multiple mutations within this region (Fig. 2*C*), expressed and purified these RADX mutants as mCherry-FLAG fusion to mutant RADX full-length proteins, and assessed which mutations affected the interaction with WT GFP-HA-RADX. RADX with residues 759 to 762 changed from NHEI to AAAA, residues 840 to 844 changed from KVEV to DVKV, and residues 847 to 849 changed from KIY to DIA reduced RADX oligomerization, while several other changes had no effect (Fig. 2*D*). To refine which residues mediate oligomerization, we mutated single residues within full-length RADX and tested their interaction with WT RADX. RADX mutants E842K, K847D, and Y849A disrupt RADX oligomerization, whereas RADX K840D retained binding (Fig. 2*E*).

**Figure 3:**
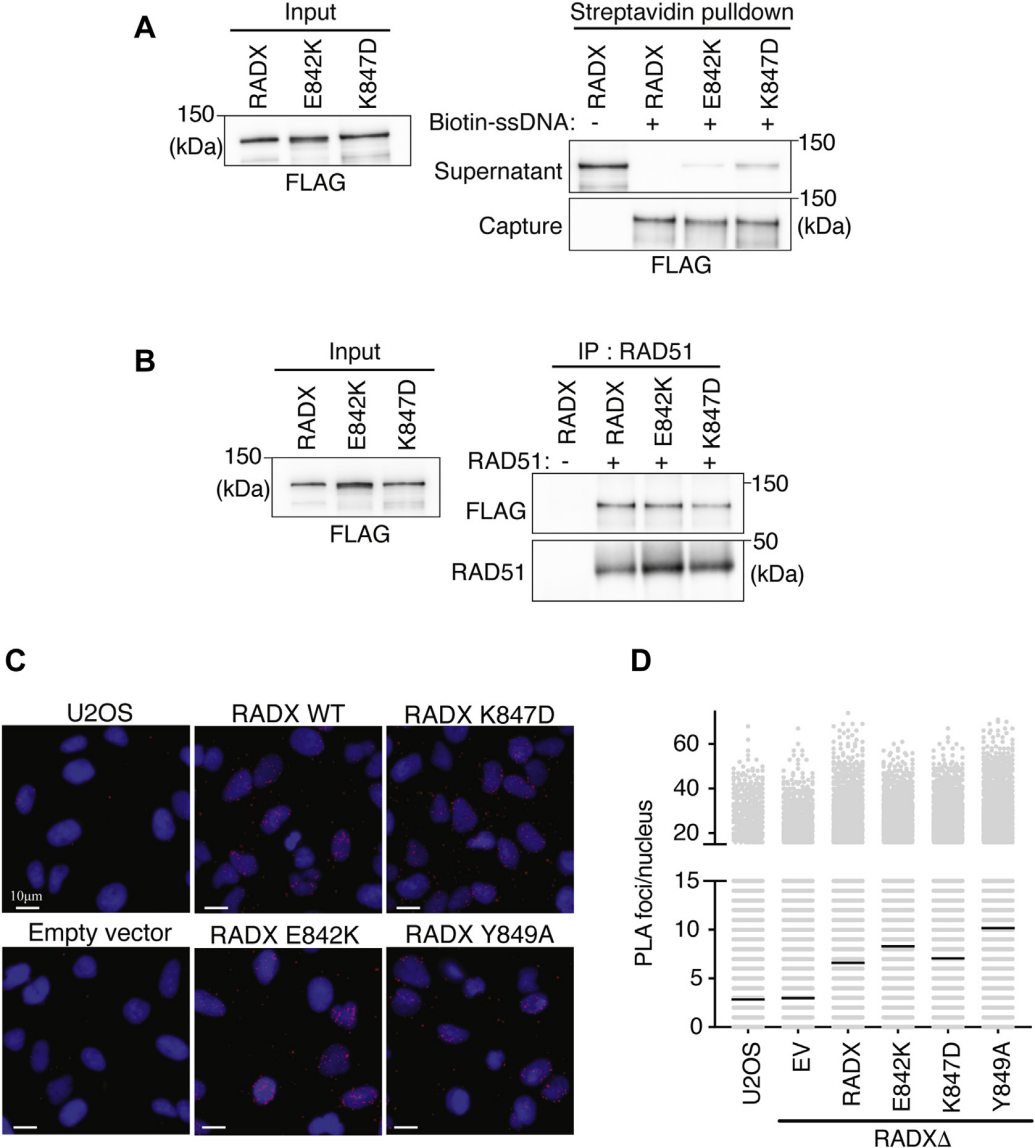
**RADX oligomerization mutants bind ssDNA and RAD51 and localize to replication forks.***A*, DNA pull-down assays of purified WT RADX or RADX mutants with ssDNA coupled to magnetic beads. *B*, direct interaction of WT RADX or RADX mutants with RAD51 was assessed by mixing purified proteins in the presence of ATP, immunoprecipitating RAD51, and immunoblotting. *C* and *D*, proximity ligation assay between FLAG-RADX and EdU after labeling cells with EdU for 20 min followed by HU for 2 h. Representative images and quantitative data are shown. EV, empty vector; HU, hydroxyurea.

E842K and K847D RADX bind to ssDNA as measured by a biotin-ssDNA pull-down assay (Fig. 3*A*), although there is a small reduction in ssDNA binding compared to WT RADX. Both RADX oligomerization mutants directly bind to RAD51 in the presence of ATP similar to WT RADX (Fig. 3*B*). Oligomerization is not required to localize RADX to stalled forks because the K847D, E842K, Y849A, and WT RADX proteins yield similar proximity ligation assay signals with nascent DNA in cells treated with HU (Fig. 3, *C* and *D*). Thus, the RADX domain 5 mutants reduce the ability of RADX to assemble into oligomers but largely retain other RADX biochemical activities and properly localize to replication forks.

### RADX oligomerization is required to maintain replication fork stability

**Figure 4:**
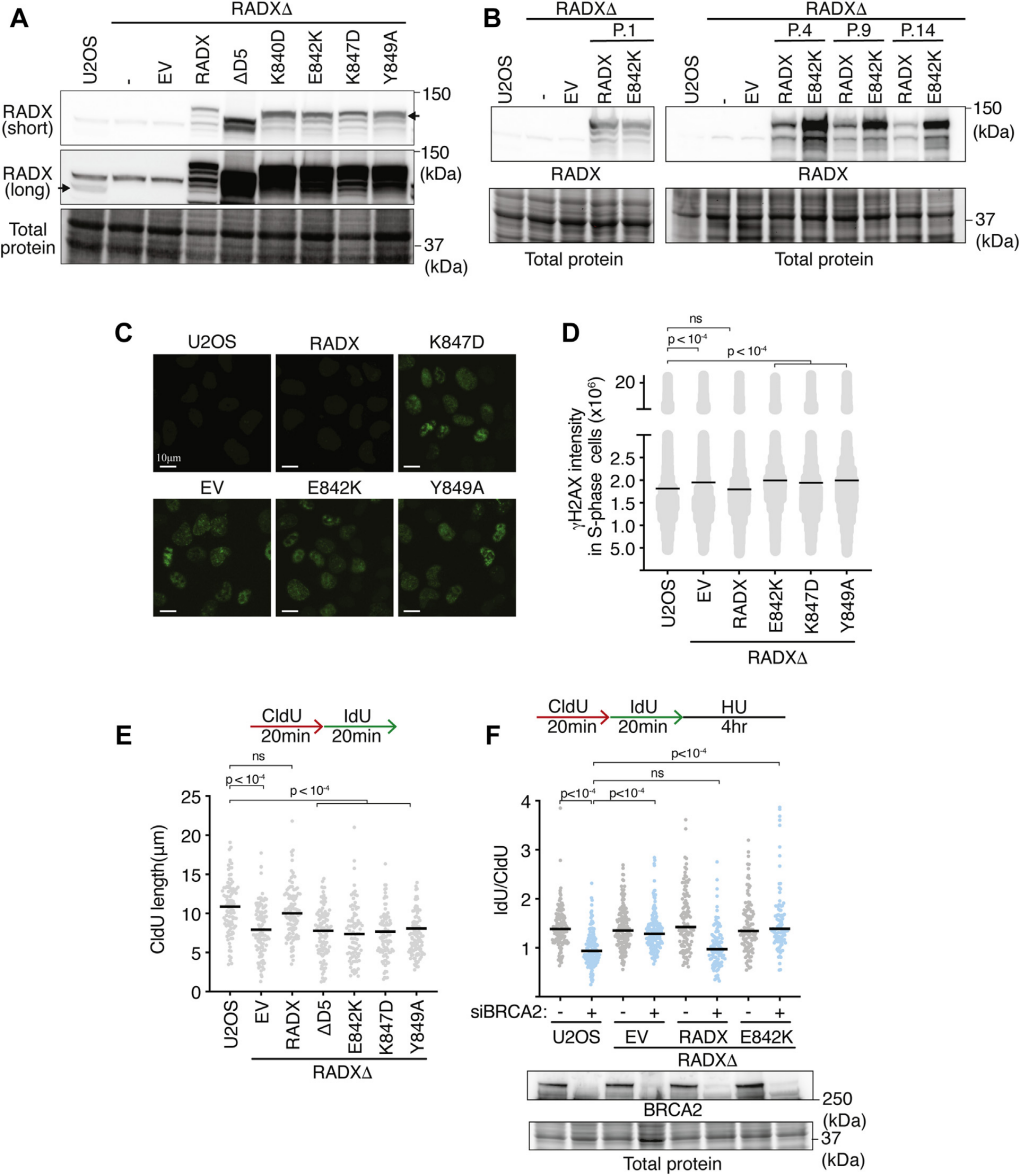
**RADX forms higher-order oligomers to maintain replication fork stability.***A* and *B*, immunoblots of U2OS or RADXΔ cells infected with lentivirus-expressing empty vector, WT RADX, or RADX mutants. For (*B*), passage number after infection and selection is indicated. *C* and *D*, γH2AX intensity in S-phase cells. *p* values were derived from a one-way ANOVA with Dunnett’s multiple-comparison test. *E*, Wild-type or RADXΔ U2OS cells complemented with WT RADX or RADX mutants were labeled with CldU (20 min) followed by IdU (20 min). CldU fiber lengths are plotted to measure elongation rate by DNA combing. A one-way ANOVA with Tukey’s multiple-comparison test was used to calculate *p* values. *F*, fork protection assays were performed in WT or RADXΔ U2OS cells complemented with WT RADX or RADX E842K and transfected with nontargeting or BRCA2 siRNA as indicated. A one-way ANOVA with Tukey’s multiple-comparison test was used to calculate *p* values. EV, empty vector; HU, hydroxyurea.

With oligomerization mutants identified, we next asked whether oligomerization is important for RADX function in cells. To this end, we complemented RADX knockout U2OS (RADXΔ) cells with retroviruses encoding either empty vector (EV), WT RADX, or RADX oligomerization mutants fused to mCherry-FLAG (Fig. 4*A*). Wild-type RADX expression in this system is rapidly attenuated as previously reported; presumably because of selection to reduce the replication problems caused by overexpression (15). However, expression levels of the RADX oligomerization mutants including E842K remained unchanged even after 14 passages (Fig. 4*B*). The observation that cells tolerate high overexpression of the E842K mutant is reminiscent of other loss-of-function mutants such as OB-2 and OB-3 mutants that interfere with ssDNA binding and RAD51 binding, respectively (15, 16).

RADXΔ cells exhibit signs of replication stress such as elevated γH2AX in the absence of exogenous DNA damage. Complementation with WT RADX rescues that phenotype; however, expression of an EV or RADX oligomerization mutants does not reduce γH2AX (Fig. 4, *C* and *D*). Moreover, RADXΔ cells exhibit a decreased rate of replication fork elongation that is also rescued by expressing WT RADX but not RADX oligomerization mutants (Fig. 4*E*). Finally, RADX inactivation rescues the nascent strand degradation observed in BRCA2-deficient cells when exposed to HU (16). Expression of WT RADX in RADXΔ cells restores nascent strand degradation upon BRCA2 inactivation. In contrast, expression of the E842K RADX oligomerization mutant does not restore nascent strand degradation in BRCA2-deficient cells (Fig. 4*F*). Taken together, these results indicate that RADX oligomerization is critical for its functions at elongating and stalled replication forks.

### Chemically induced dimerization of RADX oligomerization mutants restores RADX function

**Figure 5:**
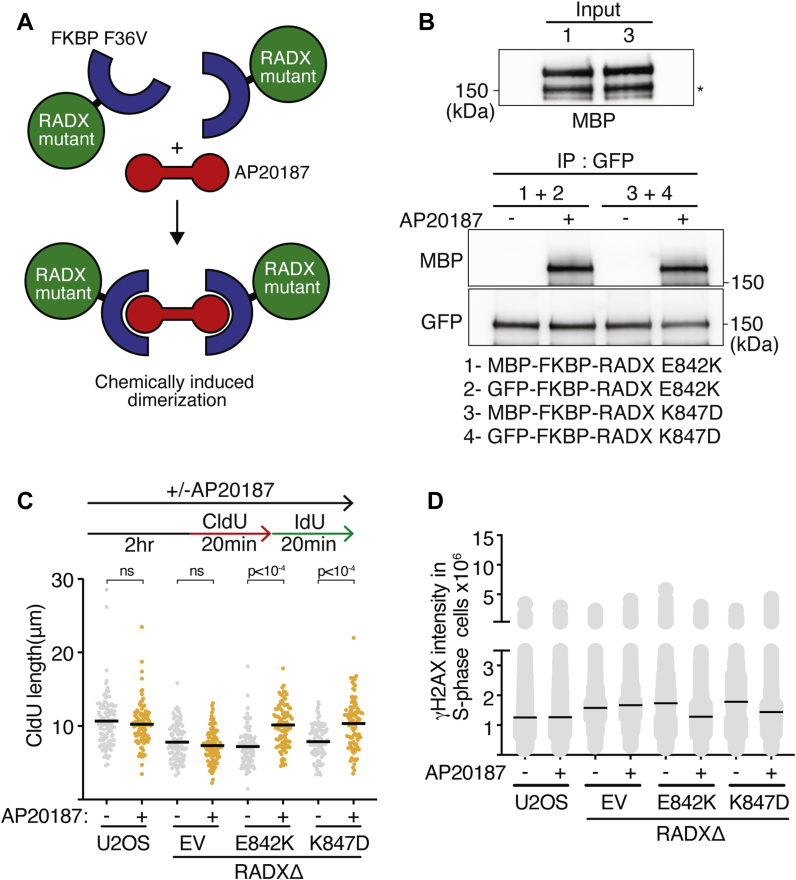
**Chemically induced dimerization restores the function of RADX oligomerization mutants.***A*, schematic of chemically induced dimerization of RADX oligomerization mutants. *B*, purified MBP-FKBP12(F36V)-FLAG fused to RADX E842K and K847D were mixed with purified GFP-FKBP12(F36V)-RADX E842K and K847D, respectively. Binding reactions were carried out with DMSO or 5 μM AP20187. GFP-immunoprecipitated proteins were separated by SDS-PAGE and immunoblotted. (∗) indicate potential degradation product. *C*, Wild-type or RADXΔ U2OS cells complemented with empty vector or RADX mutants fused to FKBP(F36V) were labeled with CldU (20 min) followed by IdU (20 min) in the presence and absence of 100 nM AP20187 as indicated. CldU fiber lengths are plotted to measure elongation rate by DNA combing. A one-way ANOVA with Tukey’s multiple-comparison test was used to calculate *p* values. *D*, γH2AX intensity was measured in EdU positive WT or RADXΔ U2OS cells complemented with empty vector or RADX mutants fused to FKBP(F36V) in the presence and absence of 100 nM AP20187 as indicated. EV, empty vector; MBP, maltose-binding protein.

We reasoned that if the RADX domain 5 mutants interfered with the ability of RADX to dimerize, then adding a heterologous dimerization domain onto RADX might be able to restore function to these mutants. To test this hypothesis, we used a version of FK506 binding protein 12 (FKBP12) harboring a F36V mutation that allows specific binding to the homo-bifunctional small molecule AP20187 to induce dimerization (20). We fused this inducible dimerization domain to the N-terminus of both MBP and GFP-tagged RADX E842K and K847D mutants and tested if AP20187 could induce dimerization (Fig. 5*A*). Indeed, MBP-tagged FKBP12-F36V-RADX E842K and RADX K847D are coimmunoprecipitated with their corresponding GFP-tagged mutant proteins only in the presence of AP20187 (Fig. 5*B*). We then asked whether the induced dimerization in cells would rescue the defects caused by the domain 5 mutants. FKBP12-F36V-RADX E842K or K847D expressing cells exhibited slow forks indistinguishable from RADXΔ cells. However, addition of AP20187 to induce RADX E842K or RADX K847D dimerization, restored fork speed to the same rate as the RADX WT expressing cells without having any effect on fork speed in the control RADXΔ cells (Fig. 5*C*). Similarly, AP20187-induced dimerization suppresses γH2AX intensity in RADXΔ cells complemented with FKBP12-F36V-RADX E842K or K847D, whereas it had no effect on RADXΔ cells complemented with EV. These results confirm that the defect observed in cells expressing RADX domain 5 mutants is because of their inability to oligomerize and that oligomerization is critical for RADX function.

## Discussion

Our findings indicate that RADX forms oligomers that are partly dependent on a self-interaction surface encoded by a predicted structured region termed domain 5 and this homo-oligomerization is essential for RADX function. While the exact stoichiometry of its oligomeric state remains unknown, chemically induced dimerization is sufficient to restore function to RADX domain 5 oligomerization mutants. Our analyses indicate that RADX oligomerization is needed to maintain fork speed, prevent fork collapse, and regulate fork protection.

In addition to domain 5, RADX likely contains at least one additional region that mediates self-association because a RADX protein containing the first three OB-fold domains can interact with full length RADX. Furthermore, disrupting the domain 5 interaction attenuates but doesn’t abolish RADX oligomerization. Additional studies will be needed to characterize this second region and determine its importance.

The exact reason RADX oligomerization is important for function remains unknown. A previous study found that RADX condenses ssDNA upon binding *in vitro* (19). It does this through forming higher order assemblies of DNA and RADX proteins. Whether this activity is important for its function in cells is unknown, but it is possible that RADX oligomerization contributes to this activity. RADX oligomerization is not required for RAD51 binding or for localization to replication forks. However, RADX oligomerization mutants exhibited a mild reduction in ssDNA binding compared to the WT RADX protein. Many other ssDNA-binding proteins such as *Escherichia coli* ssDNA-binding protein or human RPA form homo- or hetero-oligomers (21, 22). This property allows the proteins to bind ssDNA dynamically but with high affinity. It also allows them to bind DNA in multiple different conformations with different DNA-binding footprints. The small reduction in DNA binding of the domain 5 oligomerization mutants compared to the WT RADX suggests that oligomerization could mediate changes in its DNA-binding properties that ultimately result in loss of cellular functions. Further quantitative analyses of the RADX DNA-binding properties combined with structural analyses of the DNA–RADX complex will help to answer this question.

## Experimental procedures

### Cell culture

U2OS and HEK293T cells were cultured in Dulbecco's modified Eagle's medium (DMEM) with 7.5% fetal bovine serum. U2OS RADXΔ were described previously (16). Complementation of RADXΔ cells with cDNA expression vectors was completed by retroviral infection and selection for the linked neomycin resistance cassette as described (16). Plasmid and siRNA transfections were performed with polyethylenimine and Dharmafect1 (Dharmacon) respectively.

### Plasmids

The RADX cDNA was cloned into pLEGFP-HA-NLS (pTM49) or the same vector backbone with mCherry-Flag substituted for EGFP-HA (pTM39). RADX truncation mutants expressed as mCherry-FLAG-NLS fusion protein include pTM59 (2–675), pTM67 (675–855), pTM43 (675–727), and pTM44 (744–855). Mutations in *RADX* were constructed using site-directed mutagenesis and were sequence-verified. RADX mutants expressed as mCherry-Flag-NLS fusion proteins included the following: LGH to AAA (residues 748–750, pTM108), NHEI to AAAA (residues 759–762, pTM109), DV to KA (residues 765–766, pTM110), SQID to AAAK (residues 780–783, pTM111), HIVG to AAAA (residues 826–829, pTM112), KVEV to DVKV (residues 840–843, pTM113), and KIY to DIA (residues 847–849, pTM114). Single residue mutations were K840D (pTM155), E842K (pTM156), K847D (pTM157), and Y849A (pTM158).

### Protein purification

FLAG-mCherry-RADX proteins were purified from HEK293T cells. Briefly, the cells were lysed in buffer containing 50 mM Tris (pH 8.0), 150 mM NaCl, 1% Triton X-100, 10% glycerol, 1 mM MgCl_2_, 1 U/ml Benzonase, 1 mM DTT, and a complete protease inhibitor cocktail tablet (Roche). After high-speed centrifugation, the cleared lysates were incubated with Anti-FLAG M2 magnetic beads (Sigma M8823) for 2 h at 4 °C. The beads were washed four times in lysis buffer, three times in lysis buffer containing 0.7 M LiCl_2_, and finally three times in elution buffer (50 mM Tris (pH 8.0), 300 mM NaCl, 10% glycerol, and 1 mM EDTA). The bound proteins were eluted in elution buffer with 300 μg/ml 3× FLAG peptide (Sigma F4799). Eluted proteins were subjected to size-exclusion chromatography on a Superdex 200 10/300 Increase GL (GE Healthcare) in elution buffer with added protease inhibitors. For EGFP-HA-RADX, the same protocol was followed except using monoclonal anti-HA agarose beads (Sigma-Aldrich, A2095) and elution using 100 μg/ml HA peptide (Sigma I2149).

### Coimmunoprecipitation

HEK293T cells were lysed in CHAPS lysis buffer (50 mM Tris pH 7.5, 150 mM NaCl, 10% glycerol, 0.7% CHAPS, 5 μg/ml aprotinin, 5 μg/ml leupeptin, 1 mM sodium orthovanadate, 10 mM β-glycerol phosphate, 1 mM NaF, and 1 mM DTT) for 30 min on ice and cleared by centrifugation. Supernatants were incubated with EZ View Red Anti-Flag M2 affinity gel or anti-HA agarose beads for 1 to 2 h at 4 °C. Beads were then washed three times with CHAPS lysis buffer and once with Flag elution buffer (10 mM Hepes pH 7.9, 300 mM KCl, 1.5 mM MgCl2, 0.05% NP-40, and 0.5 mM DTT). The proteins bound to EZ View Red Anti-Flag affinity gel were eluted with Flag elution buffer containing 0.3 mg/ml 3× Flag peptide (Sigma-Aldrich, F4799). Immunoprecipitated proteins were separated by SDS-PAGE and detected by immunoblotting.

### Antibodies

Antibodies used in this study include Flag (Sigma F7425), HA (Biolegend, 901501), GFP (1:1000, Abcam, ab13970), MBP (custom made), RAD51 (14B4, Abcam), RADX (NBP2, Novus), BRCA2 (OP95, Calbiochem), γH2AX (JBW301, Millipore), Biotin (Cell Signaling #5597), IdU (Abcam Cat#ab6326), and CldU (BD Cat#347580).

### *In vitro* binding analysis

Green fluorescent protein-trap magnetic agarose beads (Chromotek) were washed twice in binding buffer containing (50 mM Tris pH 7.5, 250 mM NaCl, 10% glycerol, and 0.05% NP-40). Then, mCherry-FLAG-RADX, -RADX fragments, or –RADX mutants were mixed with GFP-HA-RADX fusion protein and incubated with GFP beads for 1 h at 4 °C. Supernatant was discarded and beads were washed three times with binding buffer. Finally, the bound proteins were eluted in 2× sample buffer, and immunoprecipitated proteins were separated by SDS-PAGE and detected by immunoblotting. To assess the RADX interaction with RAD51, RAD51 antibody was incubated with Protein G magnetic beads for 1 h at room temperature. The resin was washed once in binding buffer (50 mM Tris pH 8.0, 200 mM NaCl, 2 mM CaCl_2_, 2 mM ATP, and 1 mM DTT). Purified RAD51 (100 nM) was added to the beads and incubated further for 1 h at room temperature. The resin was washed twice in binding buffers before the addition of the purified RADX WT or mutant proteins for 1 h. Resin was washed twice in binding buffer before the addition of 2× SDS sample buffer and resolved by SDS PAGE and immunoblotting.

### Size-exclusion chromatography

Purified MBP-RADX was loaded onto a Superdex 200 Increase 10/300 Gl Column (GE Healthcare) equilibrated with buffer (50 mM Tris pH 7.5, 300 mM NaCl, 10% Glycerol, and 1 mM DTT). Proteins were eluted at a rate of 0.3 ml/minute and 0.5 ml fractions were collected. Equal amounts of fractions corresponding to peaks were separated by SDS-PAGE, and the proteins were detected by Coomassie staining.

### Biotin-DNA pull-down assays

Streptavidin T1 Dynabeads (Life Technologies) were washed twice in 10 mM Tris, pH 8, 1 mM EDTA and bound to biotinylated poly-dT69 ssDNA substrate at room temperature for 30 min. Beads were washed twice in TE followed with two washes in binding buffer (80 mM Tris, pH 7.5, 100 mM KCl, 5 mM MgCl_2_, 2 mM DTT, and 100 mg/ml bovine serum albumin (BSA) in RNase/DNase free water). One microliter of beads with 4 pmol of bound DNA was resuspended in binding buffer. Approximately, 500 fmol of purified protein was added to the mix and rotated at room temperature for 30 min. The supernatant was collected, and the proteins were eluted in 2× SDS sample buffer for 5 min. Both supernatants and captures were analyzed by immunoblotting.

### Immunofluorescence

Cells were plated in 96-well clear-bottom plates, incubated with media containing 10 μM EdU for 20 min, pre-extracted for 5 min on ice in 20 mM HEPES, pH 7.0, 50 mM NaCl, 3 mM MgCl_2_, 300 mM sucrose, and 0.5% Triton X-100 followed by fixation in 3% paraformaldehyde. The cells were blocked for 1 h in PBS containing 5% BSA. A click chemistry reaction was completed by the addition of 2 mg/ml sodium ascorbate, 2 mM copper sulfate, and 5 μM Alexa Fluor 647 conjugated azide in PBS for 30 min. Anti-γH2AX antibody incubation was performed for 1 h in 1% BSA in PBS followed by a 45-min incubation in secondary antibody. Nuclei were stained with a 5-min incubation with DAPI in PBS. Plates were imaged on a Molecular Devices ImageXpress system, and integrated nuclear intensity of γH2AX in EdU-positive cells was quantitated using the Molecular Devices software.

### Proximity ligation assay

Cells were plated in a 96-well plate and labeled with 10 μM EdU for 20 min and then treated with 4 mM HU for 2 h. The cells were permeabilized using 0.5% Triton X-100 solution (20 mM HEPES, 50 mM NaCl, 3 mM MgCl_2_, 300 mM sucrose, and 0.5% Triton X-100) and fixed in 3% paraformaldehyde for 5 min on ice. The cells were then incubated in 10% goat serum followed by antibodies to FLAG and anti-biotin to recognize EdU after conjugation to biotin azide by click chemistry. Proximity ligation was completed according to the manufacturer’s protocol (Sigma), and the images were obtained and quantified using a Molecular Devices ImageXpress instrument.

### DNA molecular combing

Cell were labeled with 20 μM CldU (Sigma, C6891) followed by 100 μM IdU (Sigma, l7125) with or without 100 nM AP20187 (Takara 635058). Approximately, 400,000 cells were embedded in agarose plugs, and molecular combing assay was performed as per Genomic Vision’s manufacturer instructions. The DNA was stained with antibodies that recognize IdU and CldU (Abcam Cat#ab6326, BD Cat#347580) for 1 h, washed in PBS, and probed with secondary antibodies for 45 min. Images were obtained using a 40× oil objective (Nikon Eclipse Ti). Analysis of fiber lengths was performed using Nikon Elements software.

## Data availability

All data is available in the article.

## Conflict of interest

The authors declare that they have no conflicts of interests with the contents of this article.
